# Tailless terminates the neural stem cell temporal cascade in both the optic lobe and central brain

**DOI:** 10.1242/dev.205703

**Published:** 2026-09-14

**Authors:** Jocelyn L. Y. Tang, Alex P. A. Donovan, Andrea H. Brand

**Affiliations:** Department of Cell Biology and Regenerative Medicine Institute, NYU Grossman School of Medicine, 540 1st Avenue, New York, NY 10016, USA

**Keywords:** Tailless, TLX, Neurodevelopment, Neural stem cells

## Abstract

Temporal patterning is an evolutionarily conserved mechanism to produce neuronal and glial diversity from common cells of origin during neurodevelopment. This process is controlled by a series of temporal transcription factors that are transiently expressed and drive the sequential production of specific progeny subtypes. Intermediate neural progenitors (INPs) and optic lobe neural stem cells (OL NSCs) share striking similarities in temporal factor expression despite divergent cells of origin. Tailless (Tll) is a terminal temporal factor in the visual system in OL NSCs. Its expression coincides with the termination of neurogenesis and onset of gliogenesis. Here, we report that Tll also acts as a terminal factor in *Drosophila* INPs, demonstrating functional conservation. Tll expression is activated by the preceding temporal factor Scarecrow, and represses *odd-paired* and *hamlet*. *tll* also plays a partial role in promoting gliogenesis in gliogenic NSCs. We performed genome-wide binding analysis of Tll in the OL NSCs and INPs by Targeted DamID, revealing both conserved and divergent targets, reflecting differences in regulatory outcomes. We show that temporal patterning mechanisms are conserved between different brain regions, whilst facilitating lineage-specific outputs.

## Teaser

**Summary:** Are temporal cascades conserved in different neural lineages? Combining genetic manipulation and chromatin-binding assays in *Drosophila*, this study identifies Tailless/TLX as a conserved factor in the termination of temporal cascades.

## INTRODUCTION

In *Drosophila*, the optic lobe neural stem cells (NSCs) and intermediate neural progenitors (INPs) are short-lived neural progenitors that, respectively, give rise to the visual system and adult central complex in the central brain. The NSCs of the optic lobe are derived from a symmetrically dividing neuroepithelium (NE) (Egger et al., 2010, 2007), while INPs are produced from the asymmetric division of Type II NSCs (Bello et al., 2008; Boone and Doe, 2008; Bowman et al., 2008). Remarkably, despite originating from disparate cell types, both optic lobe NSCs and INPs share a number of similarities. Both systems produce neuronal subtypes of similar molecular identity, notably the early-born Brain-specific homeobox positive (Bsh+) and late-born Twin of eyeless positive (Toy+) neurons (Bayraktar and Doe, 2013; Li et al., 2013). In both systems, gliogenesis occurs after neurogenesis, shortly before the progenitors entirely terminate proliferation. Both INPs and OL NSCs also progress through a temporal cascade (Doe, 2017). Several temporally expressed transcription factors are shared between the two systems, notably Dichaete (D), Eyeless (Ey), Homeobrain (Hbn) and Scarecrow (Scro), with the last three factors sharing conserved cross-regulatory interactions in both types of progenitors (Bayraktar and Doe, 2013; Konstantinides et al., 2022; Li et al., 2013; Tang et al., 2022a). Although the OL temporal cascade has been extensively characterised, gaps still remain in the INP temporal cascade. Most notably, transcription factors associated with the termination of the cascade and gliogenesis have not yet been identified.

Tailless (Tll) is an orphan nuclear receptor expressed in Type II NSCs (Hakes and Brand, 2020), neuroepithelium (Guillermin et al., 2015) and late optic lobe NSCs, coinciding with the termination of proliferation (Li et al., 2013). Although Tll expression has been identified in Type II NSCs and immature INPs (Hakes and Brand, 2020), expression in the INP temporal cascade has not been reported. Based on the similarities between the neural lineages, we hypothesised that Tll may also be expressed in the late INPs, mirroring the optic lobe NSCs. Furthermore, Tll was previously thought to play a role in gliogenesis in the optic lobe (Li et al., 2013), due to its expression in neuroblasts and progeny during the gliogenic period. As gliogenesis also occurs after neurogenesis in the INP lineage, we also investigated the potential role of Tll in glial specification.

In this study, we identify Tll expression in the late INPs and demonstrate its activation by the preceding temporal factor Scro. Tll represses expression of Asense (Ase), Odd-paired (Opa) and Hamlet (Ham), which are involved in lineage progression and termination of proliferation. Loss of Tll also impairs gliogenesis in the INPs. Mapping of Tll binding targets and chromatin accessibility in the four different cell types using Targeted DamID (TaDa) (Marshall et al., 2016; Marshall and Brand, 2015; Southall et al., 2013) and NanoDam (Tang et al., 2022a) demonstrates high conservation of binding targets between the INPs and optic lobe NSCs. Binding sites specific to each cell type were also identified, suggesting that the divergent roles of Tll in the INPs and optic lobe NSCs may be facilitated by different chromatin landscapes or co-factors. In sum, we present Tll as a previously unreported temporal factor in the INPs, specifying the terminal window in the temporal cascade, mirroring temporal patterning in the optic lobe NSCs.

## RESULTS

### Tailless is expressed in late INPs

**Fig. 1.**
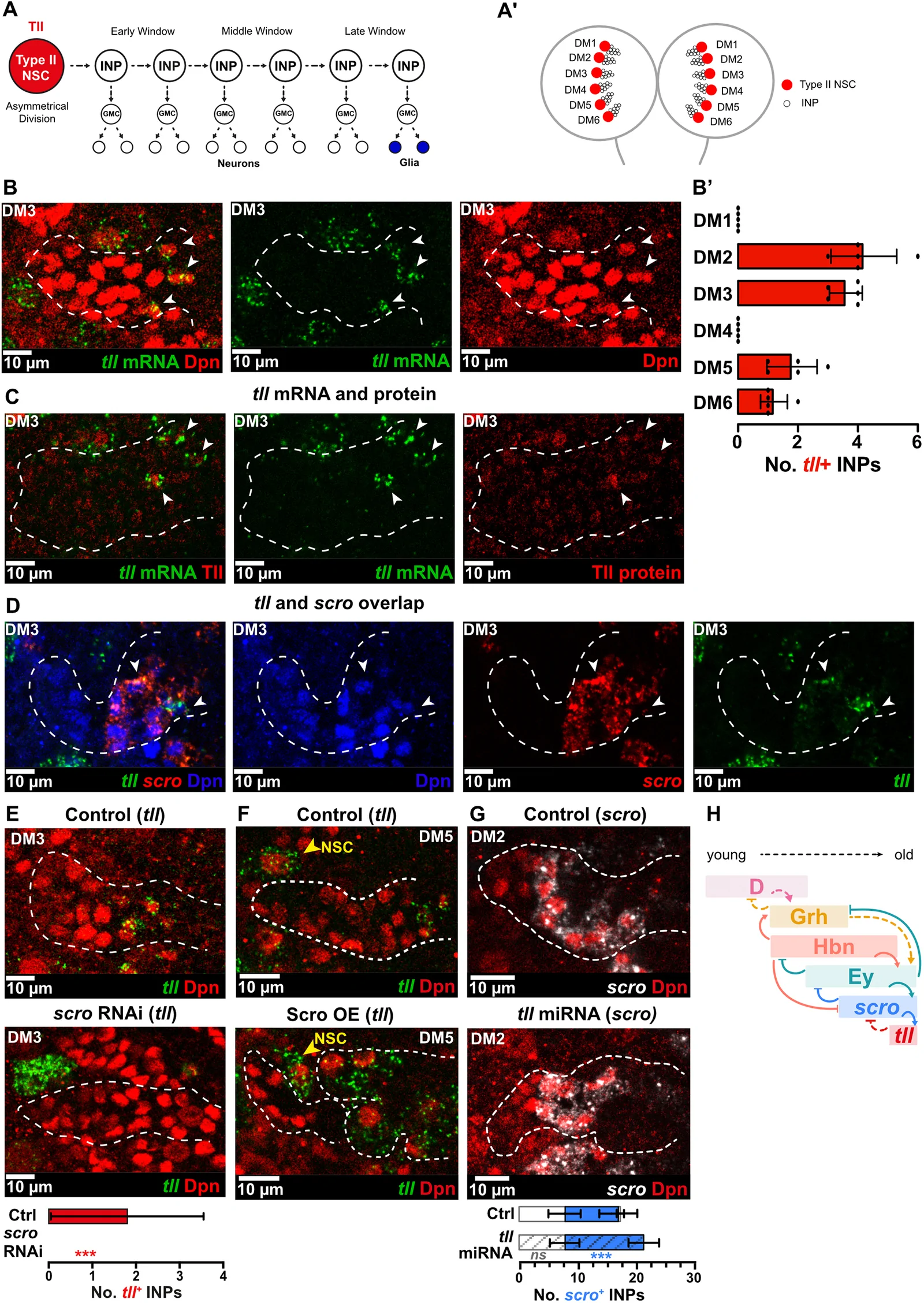
**Tailless is expressed in old intermediate neural progenitors and is activated by the temporal factor Scro.** (A) The Type II neural stem cell (NSC) lineage. Tll has been previously reported to be expressed in the Type II NSCs. The Type II NSC divides asymmetrically to produce intermediate neural progenitors (INPs), which asymmetrically divide to produce ganglion mother cells (GMCs). The GMCs divide to produce two daughter cells that differentiate into neurons or glia. (A′) The position of the six dorsal-medial (DM1-6) Type II lineages in the third instar larval brain lobes. (B) Single slice confocal images showing mRNA of *tll* (green) in a Type II lineage. Dpn (red) marks the INPs and white arrowheads indicate INPs expressing *tll* mRNA (green). The INP lineage (white dotted line) is labelled using *D*-GAL4>UAS-IVS-mCD8-RFP (which labels mature INPs and daughter cells). (B′) Quantification of *tll*-expressing INPs across each dorso-medial (DM) lineage, *n*=6 brains. (C) Single slice confocal images showing overlap of *tll* mRNA (green) and Tll protein (red) in an old INP (white arrowheads within dotted line). The INP lineage is labelled using *D*-GAL4>UAS-IVS-mCD8-RFP. (D) Single slice confocal images showing the overlap of *scro* (red) and *tll* (green) mRNA expression in the INPs (marked by Dpn in blue). The INP lineage (white dotted line) is labelled using *D*-GAL4>UAS-IVS-mCD8-RFP. Arrowheads indicate INPs that are *tll*-positive. (E) Loss of *scro* expression results in a complete loss of *tll*-positive INPs (*tll* mRNA in green, Dpn in red; single slice confocal image). White dotted line indicates lineage labelled by *D*-GAL4>UAS-IVS-mCD8-RFP (control versus UAS-*scro*-RNAi). The numbers of *tll*-positive INPs are shown as an average across DMs 1-6. Grayscale images of separate channels are shown in Fig. S1B and quantifications split by lineage in Fig. S1C, *n*=6 brains. (F) Overexpression of Scro leads to ectopic expression of *tll* (green) within the lineage (dotted line) and in INPs (Dpn in red). The INP lineages (labelling all INPs and all progeny produced) were labelled with *erm*-GAL4, Act(y+STOP)-GAL4, tubGAL80^ts^>UAS-GFP (control versus UAS-Scro). (G) Single slice confocal images showing the effect of *tll* knockdown on *scro* expression. Number of *scro*-negative (grey) and *scro*-positive (blue) INPs are shown as an average across DMs 1-6. Single channel images are shown in Fig. S1E and quantifications split by DM lineage are shown in Fig. S1F, *n*=5 brains. (H) Summary diagram of the regulatory interactions in the INP temporal cascade identified in this study and by Tang et al. (2022a,b), and Bayraktar and Doe (2013). All larval brains were staged to 96 h after larval hatching (ALH). Data are mean±s.d. Statistical significance was tested with the Mann–Whitney *U*-test: ***P<0.001 and ns (*P*>0.05).

The Type II NSC divides asymmetrically to produce INPs, which in turn divide asymmetrically to produce ganglion mother cells (GMCs) that give rise to neurons (Fig. 1A). The INPs transition through their own temporal cascade before terminating proliferation. Previous studies have identified expression of Tll in the neuroepithelium, optic lobe NSCs (Guillermin et al., 2015; Li et al., 2013) (Fig. 1A) and the Type II NSCs (Hakes and Brand, 2020). Due to the high degree of conservation in both lineages (Tang et al., 2022a,b), we hypothesised that Tll would be expressed in the oldest INPs.

To establish whether Tll plays a role in the temporal cascade of INPs, we first examined expression of both the transcript and protein. With *in situ* hybridisation chain reaction (HCR), we observed *tll* transcript in the Type II NSCs, as previously reported (Hakes and Brand, 2020) but also in the oldest INPs (Fig. 1B, Fig. S1A). The *Drosophila* larval brain is composed of two symmetrical brain lobes, with each lobe harbouring 6 dorsal-medial (DM) Type II lineages, each named in DM1-6 from anterior to posterior (Fig. 1A′). *tll*+ INPs were detected in all DM lineages except DMs 1 and 4 (Fig. 1B′), with the most *tll*+ INPs detected in the DM2 lineage. Tll protein was also present in INPs that expressed the transcript (Fig. 1C). In the context of the temporal cascade, *tll* expression overlaps with the late temporal factor *scro*, though the onset of *scro* expression precedes Tll (Fig. 1D). This expression pattern is similar to that observed in optic lobe NSCs (Konstantinides et al., 2022; Tang et al., 2022a,b).

### Tll is a terminal temporal factor activated by Scro

Based on the expression pattern of *tll* relative to *scro*, we postulated that Scro could activate the expression of *tll* in old INPs. To test this, we reduced Scro levels by driving the expression of a *scro* RNAi construct (Tang et al., 2022a,b) only in INPs using *D*-GAL4 (GMR12E09-GAL4 (Bayraktar and Doe, 2013). Knockdown of *scro* results in the complete loss of *tll* expression in old INPs (Fig. 1E, Fig. S1B,C). Conversely, misexpression of Scro results in ectopic expression of *tll* in the INP lineage (Fig. 1F, Fig. S1D). Knockdown of *tll* using a *tll* miRNA (Lin et al., 2009) also leads to prolonged *scro* expression (Fig. 1G, Fig. S1E,F). These results confirm that Scro activates *tll* expression in a time-dependent manner and *tll* exerts a repressive effect on *scro* expression, a regulatory interaction commonly observed between transcription factors in temporal cascades (Fig. 1H).

**Fig. 2.**
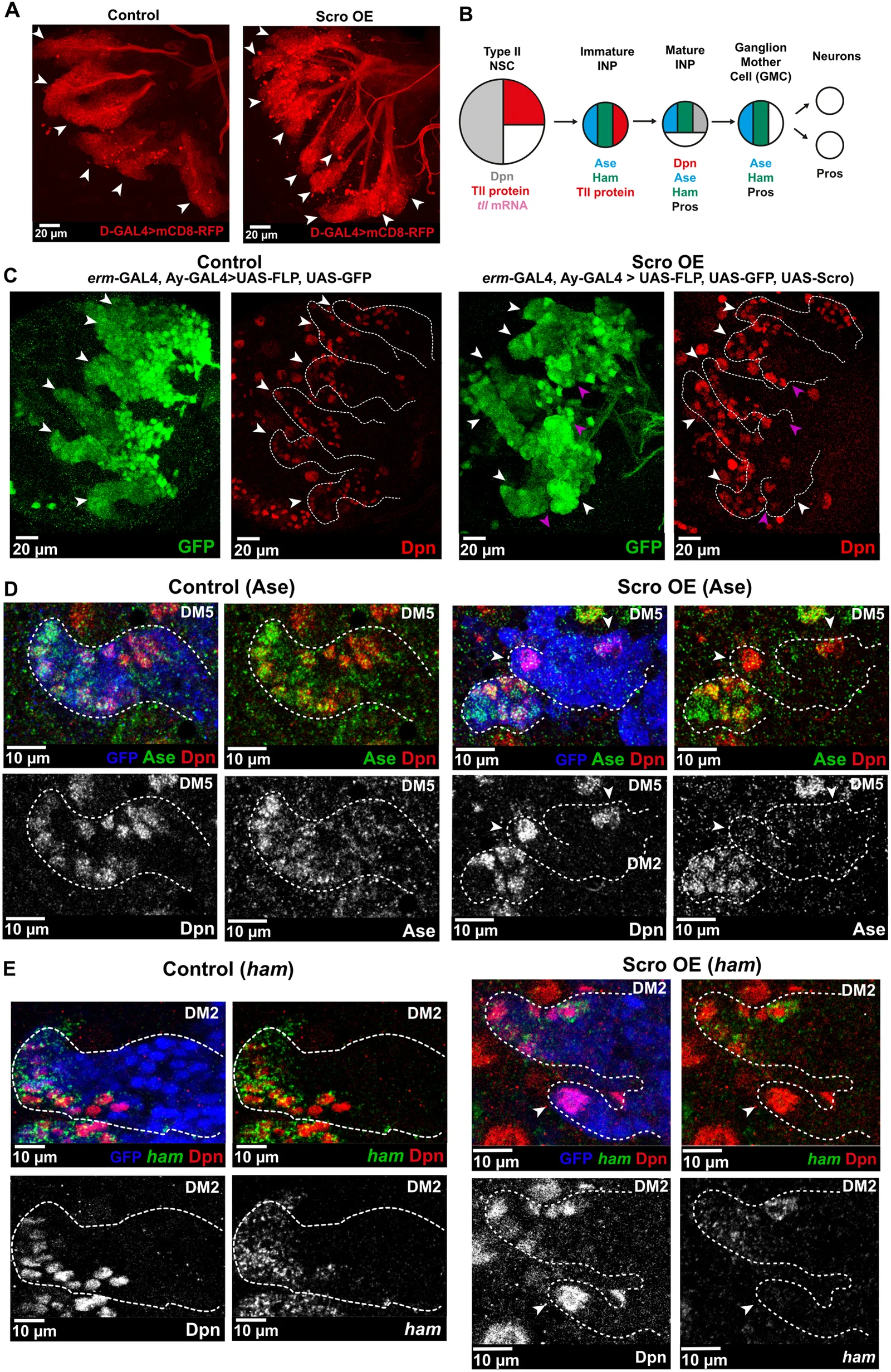
**Misexpression of Scro in young intermediate neural progenitors (INPs) causes ectopic Tll expression, resulting in some INPs reverting to Type II neural stem cells and in multiple lineages.** (A) Maximum intensity projection images showing the supernumerary lineage phenotype. White arrowheads indicate individual lineages, labelled with D-GAL4>UAS-IVS-mCD8-RFP. (B) The expression of marker proteins Deadpan (Dpn), Tailless (Tll), Asense (Ase), Hamlet (Ham) and Prospero (Pros) in the Type II neural stem cell (NSC) lineage. (C) Maximum intensity confocal images showing the variable supernumerary lineages as labelled by *erm*-GAL4, Ay-GAL4>UAS-GFP (which labels all INPs and subsequent progeny produced). White arrowheads indicate DMs1-6; magenta arrows indicate possible supernumerary lineages in the Scro overexpression condition, populated by larger Dpn-positive (red) cells and higher GFP intensity (green). (D) Single slice confocal image showing Ase expression in Type II lineages upon Scro overexpression. In the control, Ase (green) is expressed mature INPs marked by Dpn (red) in the lineage (dotted line). When Scro is overexpressed, there are some Dpn-positive cells within the lineage that are Ase negative. The nuclear size suggests larger cells, suggesting that they have reverted to a Type II NSC fate. Arrowheads indicate instances of Type II NSC-like cells within the INP lineage, suggesting cell fate reversion. (E) Single cell confocal image showing *ham* expression in Type II lineages. When Scro is overexpressed, large Dpn-positive cells (white arrowheads) within the lineage also lack *ham* (green) expression, further supporting the observation that the cell has taken a Type II NSC identity. All larval brains were staged to 96 h after larval hatching (ALH) with *n*=6 brains.

A previous study has shown that aberrant expression of Tll in younger INPs results in the reversion to an NSC fate (Hakes and Brand, 2020). Interestingly, misexpression of Scro in INPs led to the appearance of supernumerary lineages (Fig. 2A). Normally six Type II NSCs correspond to six DM lineages, whereas misexpression of Scro results in the presence of eight to ten lineages per brain lobe. This phenotype was also previously observed upon simultaneous knockdown of the transcription factors Ham and Opa (Abdusselamoglu et al., 2019), both of which are not temporally expressed (Eroglu et al., 2014). Functional manipulation of Opa leads to changes in the temporal cascade and supernumerary INPs (Abdusselamoglu et al., 2019). Unlike Opa, loss of Ham results in INP reversion back to a Type II NSC fate (Rives-Quinto et al., 2020). These observations led us to question whether the activation of Tll expression by Scro also leads to a reversion of some INPs back to the Type II NSC fate.

To investigate changes in cell identity within INP lineages, we examined the expression of transcription factor Ase, which is present only in mature INPs and not in Type II NSCs (Fig. 2B) (Hakes and Brand, 2020). We performed lineage tracing of INPs using *earmuff* (*erm*)-GAL4 (GMR9D11-GAL4) and Ay-GAL4 (which immortalises expression) while ectopically expressing Scro (Fig. 2C). Normally, mature INPs that are Dpn+ also express Ase. As expected, all lineage traced INPs in the control condition were Ase+ (Fig. 2D). However, misexpression of Scro resulted in the appearance of some Dpn+ Ase− cells in the lineage that exhibited a larger nuclear size (Fig. 2D), suggesting a Type II NSC identity. To further elucidate the identity of the Dpn+ Ase− cells, we also observed that expression of *ham* was decreased. In the control condition, *ham* is expressed throughout the INP lineage (Fig. 2E). In sum, misexpression of Scro in young INPs results in the activation of Tll, which results in the reversion of a subset of INPs back to a Type II NSC fate. These results demonstrate that Scro activates Tll in the late INP temporal cascade.

### Tll represses the expression of INP identity and lineage progression factors Ham and Opa

**Fig. 3.**
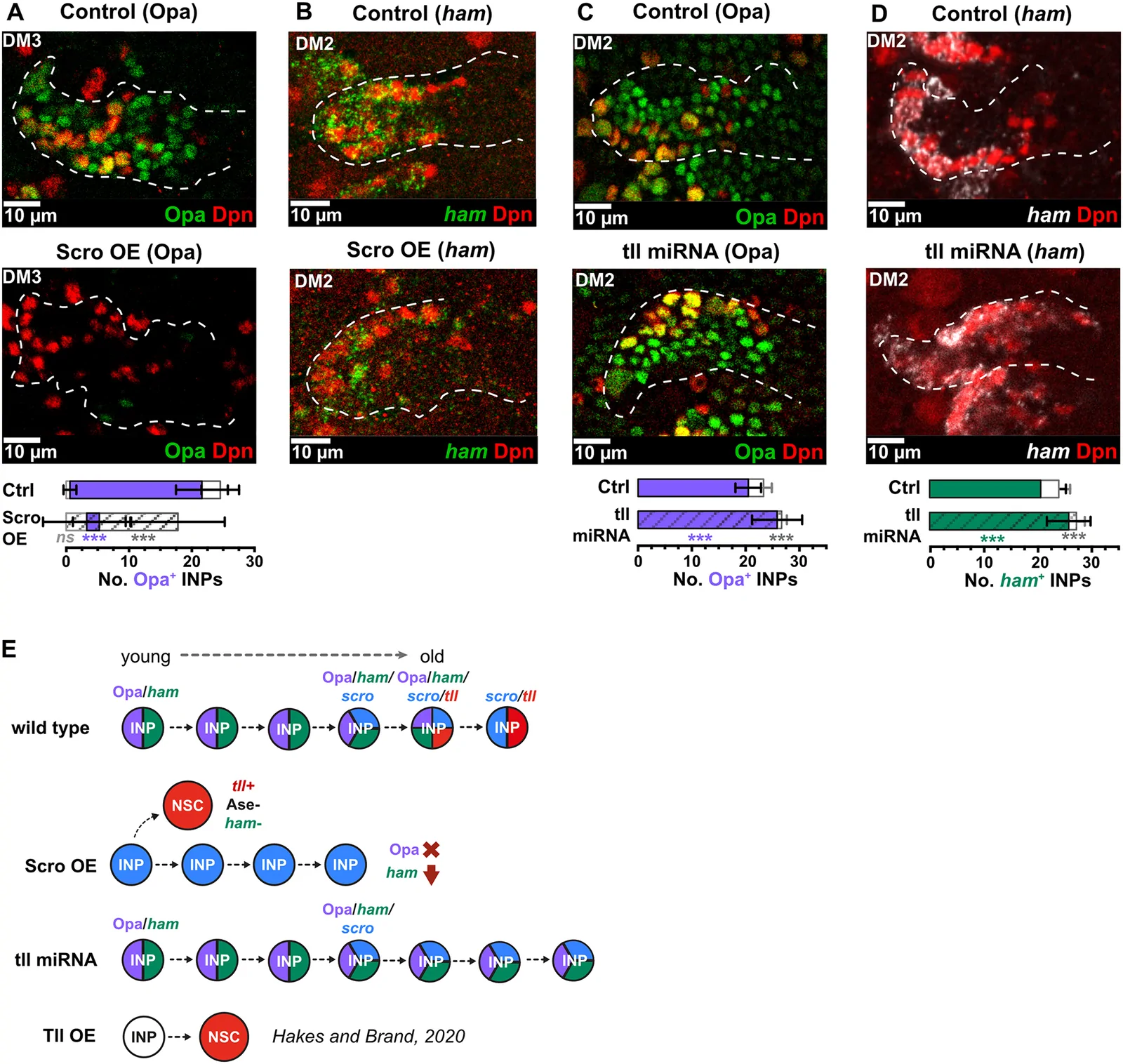
**Ham and Opa are repressed upon Scro misexpression and by Tll.** (A) Single slice confocal images showing that misexpression of Scro leads to severe loss of Opa (green) expression. Lineages (dotted lines) labelled by *D*-GAL4>UAS-IVS-mCD8-RFP. Number of Opa-positive cells out of the total number of intermediate neural progenitors (INPs) are shown as an average across DMs 1-6. Grayscale images of separate channels are shown in Fig. S2A and quantifications split by lineage in Fig. S2B. *n*=5 brains. (B) Single slice confocal images showing the expression of Scro leads to a reduction of *ham* (green) expression in INPs (red). Lineages in this experiment are labelled (dotted lines) by *erm*-GAL4, Ay-GAL4>UAS-GFP. Grayscale images of separate channels are shown in Fig. S2C, *n*=5 brains. (C) Tailless knockdown leads to an expansion of the Opa (green) window and an increase in the number of INPs. Lineages (dotted lines) are labelled by *D*-GAL4>UAS-IVS-mCD8-RFP. The number of Opa-positive cells out of the total number of INPs are shown as an average across DMs 1-6. Grayscale images of separate channels are shown in Fig. S2D and quantifications split by lineage in Fig. S2E, *n*=5 brains. (D) Tailless knockdown leads to increased *ham* (green) expression. Dotted lines outline the Type II lineage. INPs are marked by Dpn (red). Lineages (dotted lines) labelled by *D*-GAL4>UAS-IVS-mCD8-RFP. The number of *ham*-positive cells out of the total number of INPs are shown as an average across DMs 1-6. Grayscale images of separate channels are shown in Fig. S2F and quantifications split by lineage in Fig. S2G, *n*=5 brains. (E) Schematic summarising the effects of Scro overexpression, *tll* knockdown and Tll overexpression on the temporal windows of Opa, *ham*, *tll* and *scro*. All larval brains were staged to 96 h after larval hatching (ALH). Data are mean±s.d. Statistical significance was tested with the Mann–Whitney U test: ***P<0.001 and ns (*P*>0.05).

The supernumerary lineage phenotype observed upon Scro misexpression (Fig. 2A,B) was also observed upon simultaneous knockdown of the transcription factors *ham* and *opa* (Abdusselamoglu et al., 2019). This suggests that there are likely regulatory relationships between Scro, Tll, Ham and Opa, affecting INP cell identity and lineage progression. First, our results demonstrated that Scro activated *tll* and repressed *ham*. This led us to hypothesise that Scro represses *opa*, as it does with *ham* (Fig. 2E). Opa is normally expressed in most INPs, except for the oldest in the lineage (Fig. 3A). Ectopic Scro expression severely reduced Opa expression in the INPs (Fig. 3A, Fig. S2A,B), with effects on the earlier temporal factors D and Grh (Fig. S2H-K). In INPs that have not reverted to a Type II NSC fate, *ham* expression is reduced upon Scro overexpression (Fig. 3B, Fig. S2C). Scro therefore represses both *ham* and *opa*.

We then wanted to test whether the repressive effects of Scro on *ham* and *opa* were mediated through Tll. Interestingly, the expression of Opa and *ham* is absent in Tll+ INPs (Fig. 3C,D). As misexpression of Tll in the INPs leads to an over-proliferation of cells that have reverted to Type II NSCs (Hakes and Brand, 2020), we investigated the effects of knocking down *tll* by with *tll*-miRNAs (Lin et al., 2009). Ham and Opa are normally expressed in older INPs but are switched off in the oldest cells (Abdusselamoglu et al., 2019; Eroglu et al., 2014). *tll* knockdown in INPs results in the expansion of both the Opa and *ham* expression window (Fig. 3C,D, Fig. S2D-G). *tll* knockdown also resulted in an increase in INP number, though this increase was not as dramatic as that we observed upon *scro* knockdown (Tang et al., 2022a,b). These results suggest that Scro could repress *ham* and *opa* through the activation of Tll. However, the possibility remains that this may not be the sole regulatory interaction that mediates the effects of Scro on *ham* and Opa expression.

### Tll-binding targets are broadly conserved between cell types in the larval CNS

**Fig. 4.**
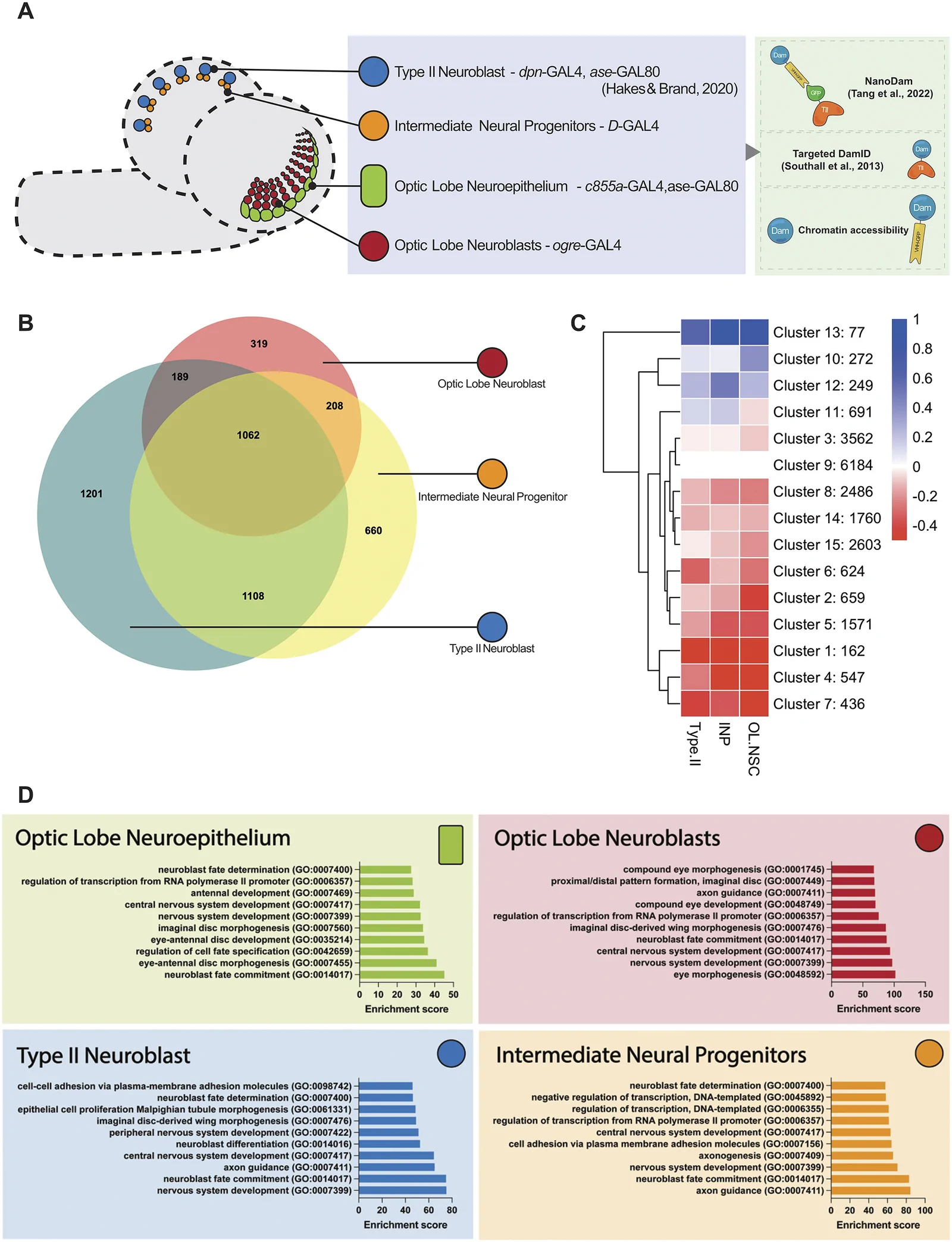
**Targeted TaDa and NanoDam to profile Tll binding in four cell types.** (A) Methods used for cell type-specific DamID profiling of Tll binding in the *Drosophila* larval brain. (B) Venn diagram showing overlapping and separate binding targets of Tll identified in the optic lobe neuroblasts (red), intermediate neural progenitors (yellow) and Type II neuroblasts (blue). (C) K-means clustering of Tll binding at all genes. (D) Gene ontology analysis for Tll-bound genes in each cell type.

As both the temporal expression profile of Tll and its regulatory relationships with other factors in the temporal cascade are conserved between INPs and optic lobe NSCs, we next aimed to ascertain whether the binding targets of Tll are also conserved. To map and compare Tll-binding targets genome-wide, we utilised Targeted DamID (TaDa) to profile INPs and integrated this with previously published TaDa data from Type II NSCs (Hakes and Brand, 2020). To profile Tll targets in the neuroepithelium and optic lobe NSCs, we took advantage of an endogenously tagged GFP protein trap of Tll in combination with NanoDam (Tang et al., 2022a,b). To ensure cell type specificity, we used a combination of cell type-specific GAL4 drivers and GAL80s to refine the expression of DamID tools (Fig. 4A).

We hypothesised that the binding profiles of Tll would be similar between the INPs and optic lobe NSCs, while the Type II NSCs and neuroepithelium profiles would be more distinct. As expected, Tll binding in the INPs and optic lobe NSCs overlapped substantially (1270 genes), with a smaller number of INP or optic lobe NSC-specific targets being identified (319 and 680, respectively) (Fig. 4B). Interestingly, the Type II NSC also shared many binding sites with the INPs and optic lobe NSCs (2170 and 1251 respectively), with clustering of Tll binding intensities over all genes showing strong correlation between all cell types (Fig. 4C). As expected, the neuroepithelium had a significantly different Tll binding profile in comparison to the other three cell types. Despite these differences, bound genes in all cell types were enriched for gene ontology terms linked to neuroblast fate commitment and central nervous system development (Fig. 4D). In the optic lobe neuroepithelium and optic lobe neuroblasts, there was a specific enrichment for gene ontology terms related to eye development (Fig. 4D). We next genome-wide performed analysis for motifs enriched within significant Tll-binding sites (Table S1). This analysis revealed differences in significantly enriched motifs between lineages, suggesting that different co-factors may serve to diversify aspects of Tll function (Table S1). In Type II NSCs, a sequence motif was identified for *buttonhead* (*btd*), which is necessary for the generation of mature INPs (Komori et al., 2014; Xie et al., 2014). In the INPs, motifs for other temporal factors such as *opa* and *homeobrain* (*hbn*) were identified, while in optic lobe NSCs, *D* was identified, where it defines the late temporal window, overlapping with Tll expression (Konstantinides et al., 2022). This overlap of Tll- and Opa-binding sites is intriguing, as it suggests that Tll may be acting both to repress expression of Opa and its target genes, conceivably to sharpen the conclusion of the Opa temporal window. Taken together, this shows that although Tll plays a similar regulatory role during the temporal cascades of these cell types, it may also play context-specific roles to act in a tissue-specific manner.

**Fig. 5.**
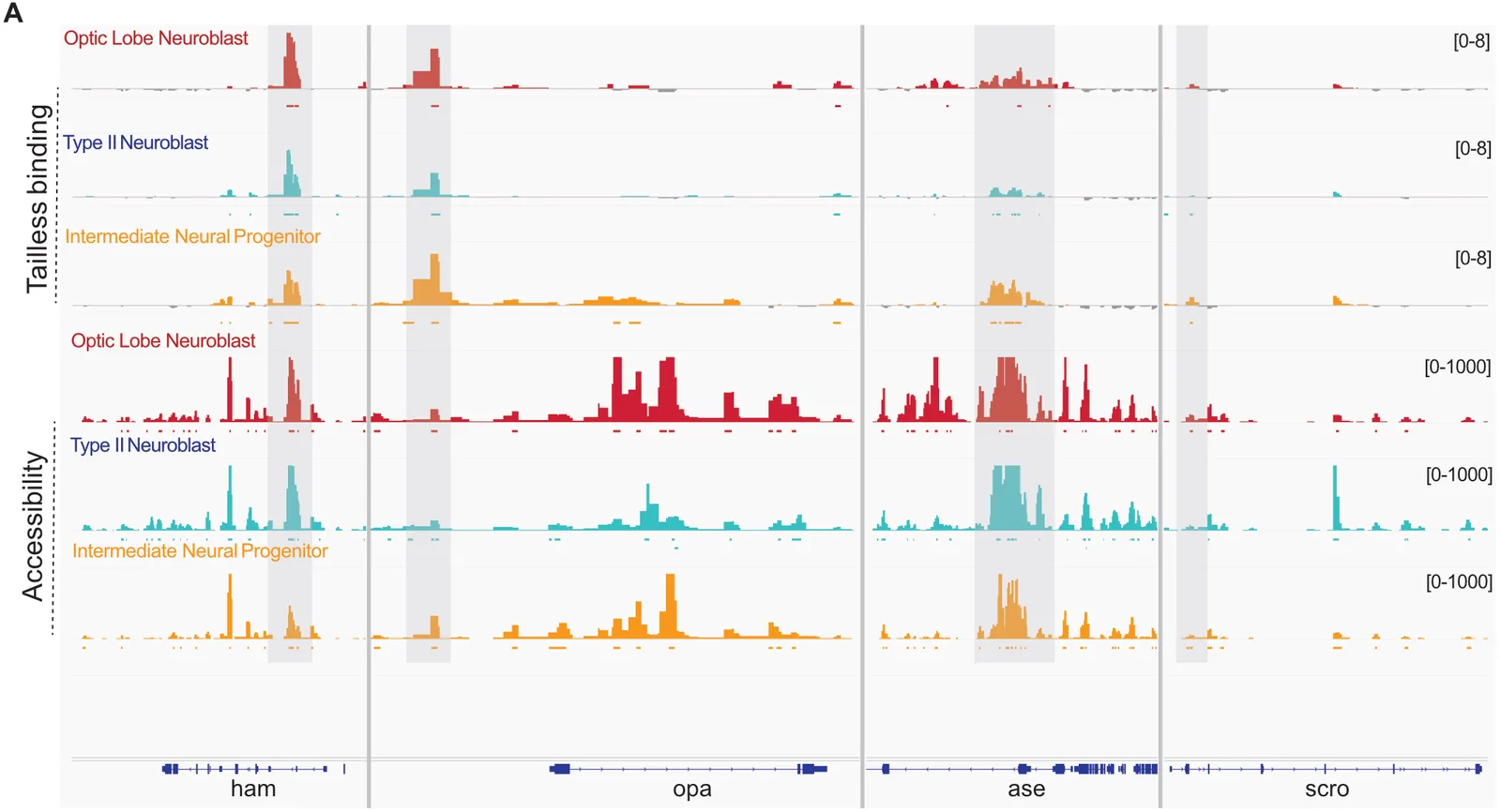
**Targeted DamID of Tll binding.** (A) Tll binding and chromatin accessibility at the *ham*, *opa*, *ase* and *scro* loci in optic lobe neuroblasts (red), Type II neuroblasts (blue) and intermediate neural progenitors (yellow).

Examination of specific loci shows that there is strong binding around the promoter regions of *ham*, *opa* and *ase* in the INPs, optic lobe NSCs and the Type II NSCs (Fig. 5A). Low level Tll binding was also detected at the *scro* locus in all three cell types (Fig. 5A). These similarities in binding, taken together with the functional experiments strongly suggest that Tll acts as a transcriptional repressor in the Type II NSC and as a canonical temporal transcription factor in the optic lobe NSCs and INPs.

### Tll promotes gliogenesis in INPs

**Fig. 6.**
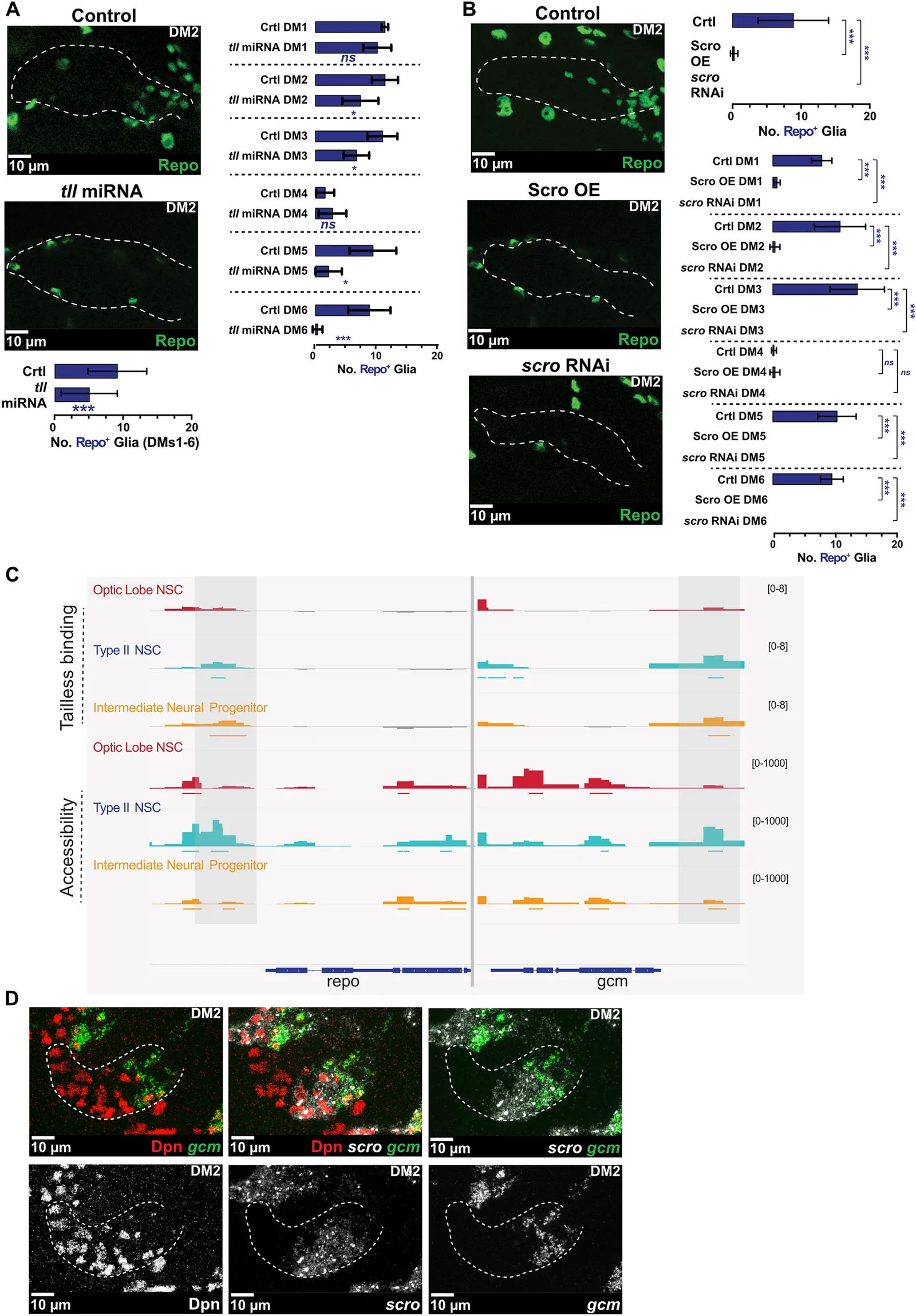
**Scro and Tll affect gliogenesis.** (A) Single slice images showing that both *tll* knockdown and Tll overexpression reduce number of Repo-positive glia (green) produced. Graphs show the number of glia averaged over all DM lineages and shown for each DM lineage separately. All larval brains were staged to 96 after larval hatching (ALH), *n*=6 brains. (B) Single-cell confocal images showing that both the overexpression and knockdown of Scro lead to a complete loss of Repo-positive glia (green) in all DM lineages. (C) Tll binding tracks of the *repo* and *gcm* loci in Type II neural stem cells (NSCs), intermediate neural progenitors (INPs) and optic lobe NSCs. (D) Single cell confocal images showing the expression patterns of *gcm* (green) and *scro* (white) in a Type II lineage (dotted lines, labelled with D-GAL4>UAS-IVS-mCD8-RFP). *gcm* expression is detected in INPs (Dpn in red) and other cell types, beginning after *scro* expression. Data are mean±s.d. Statistical significance was tested with the Mann–Whitney *U* test: ***P<0.001, *P<0.05 and ns (*P*>0.05).

Aside from being a terminal temporal factor in both the INPs and optic lobe NSCs, Tll expression also coincides with the onset of gliogenesis (Bayraktar and Doe, 2013; Li et al., 2013). Previous work in the optic lobe has shown that Tll expression overlaps with the oldest gliogenic NSCs (Li et al., 2013). However, a more recent study has suggested that Tll does not impact gliogenesis (Zhu et al., 2022). We therefore examined the effect of Tll on gliogenesis by performing lineage tracing coincidentally with expression of *tll* miRNA. Lineage tracing of INPs, while knocking down *tll* expression, showed a reduction but not complete removal of glia [marked by Reversed polarity (Repo)] (Fig. 6A). This is true for all lineages except DM1 and DM4, where *tll* is not expressed in late INPs. This suggests that Tll is not necessary for gliogenesis but does have the capacity to exert an effect when it is expressed. We also examined the effect of the Scro on gliogenesis, as it is the upstream activator of Tll. Both loss and misexpression of Scro led to a complete loss of glia in the lineages (Fig. 6B), akin to what was observed in previous experiments manipulating levels of Dichaete, Grainyhead and Eyeless (Bayraktar and Doe, 2013). The severe phenotype of Scro in comparison to Tll suggests that Scro regulates other downstream gliogenic factors in addition to Tll.

Tll binding at the *repo* locus is significant in both the Type II NSC and INPs but not in the optic lobe NSCs (Fig. 6C). In the optic lobe, Tll is expressed in the oldest optic lobe NSCs and some of the glial progeny (Li et al., 2013). However, it was shown that *glial cells missing* (*gcm*), rather than Tll primarily regulated gliogenesis in this context (Zhu et al., 2022). Tll binding is absent at the *gcm* locus in the INPs and optic lobe NSCs but present in the Type II NSCs (Fig. 6C). mRNA expression of *gcm* is detected in the INPs, preceding the expression of *tll,* but not the Type II NSCs (Fig. 6D). Together, this suggests that Tll regulates *repo* in the INPs but not *gcm* expression, whereas in the Type II NSC it regulates both. Although Tll may exert influence on gliogenesis in INPs, other factors such as *gcm* may be more crucial. This is also reflected in the optic lobe NSCs, where *gcm* exerts a strong effect on gliogenesis (Zhu et al., 2022).

## DISCUSSION

Temporal patterning is a key mechanism through which neuronal and glial diversity is generated in the developing brain. This is achieved by the establishment of temporal windows, typically defined by transiently expressed temporal factors that directly regulate progeny subtype specification in a restricted manner (Holguera and Desplan, 2018). Temporal patterning has been observed in the vertebrate retina (Young, 1985), during the generation of cortical projection neurons (Alsiö et al., 2013; Caviness, 1982; Telley et al., 2019) and spinal interneurons (Stam et al., 2012). Temporal factors typically cross-regulate each other, repressing the expression of those from the previous window and inducing those expressed in the subsequent window, producing a tightly constrained temporal cascade. These temporal cascades are also regulated by the initiation and termination of NSC proliferation, which must occur in a timely manner to generate and maintain appropriate cell numbers. Temporal cascades have been extensively studied in *Drosophila*, the two most prominent being in the embryo and larval optic lobe (Doe, 2017). A less well understood temporal cascade exists in the INPs of the central brain (Bayraktar and Doe, 2013). However, more recent work has shown the first case of regulatory conservation between temporal cascades of the larval optic lobe and INPs (Tang et al., 2022a,b).

**Fig. 7.**
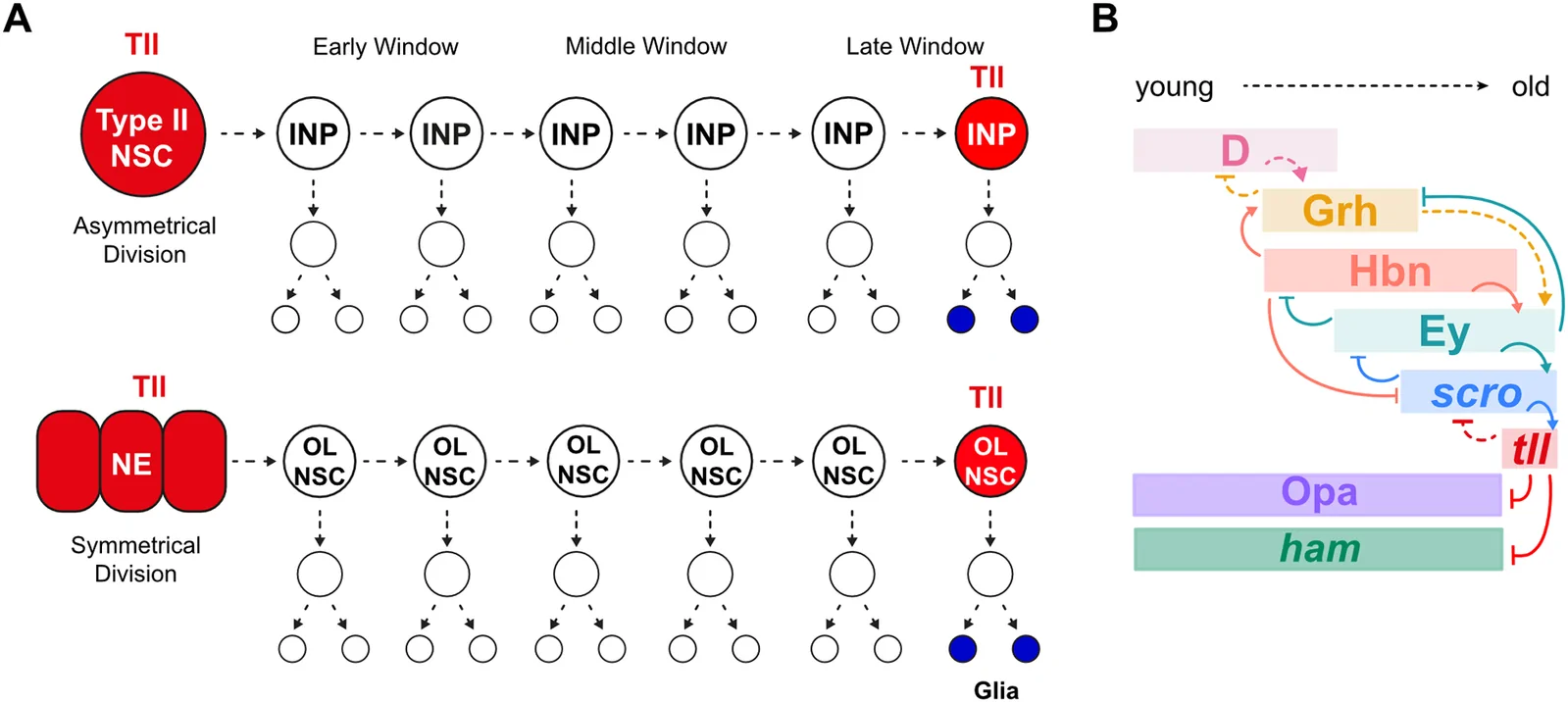
**Tll marks the termination of the temporal cascade in both intermediate neural progenitors and OL neural stem cells.** (A) Summary diagram showing similarities in Tailless expression in both neural lineage systems. Expression of Tailless in the Type II neural stem cells (NSCs) and neuroepithelium has roles in cell fate and identity, whereas Tailless expression in intermediate neural progenitors (INPs) and OL NSCs (short-lived progenitors) marks both the gliogenic progenitors and the termination of the temporal cascade. (B) Expression patterns of temporal and lineage progression factors across the INP lifespan.

Tll expression has previously been described exclusively in Type II NSCs and immature INPs. Here, we describe its expression in late INPs, acting as a terminal temporal factor in the temporal cascade. This reflects the end of the temporal cascade in the optic lobe, further capturing the striking similarities between the neural lineages of the central brain and visual system (Fig. 7A). This shared regulatory logic between lineages in the developing central nervous system raises the intriguing possibility of a core transcriptional clock that could govern neural stem cell temporal progression, upon which lineage specific information can be layered.

The identification Tll expression in late INPs enabled the study of its regulatory interactions with transcription factors involved in the temporal cascade. We found that Tll is activated by *scro* (Fig. 7B), which is expressed in the previous window of the temporal cascade. Tll, as the subsequent temporal factor, represses the expression of *scro* (Figs 1E, 7B). The activation of the subsequent temporal factor by the previous temporal factor, and the repression of the previous temporal factor by the subsequent temporal factor represents an example of a canonical interaction characteristic of temporal cascades. This facilitates the sequential activation of temporal factors, but also regulates the duration of each temporal window. In optic lobe NSCs, *scro* expression does overlap with Tll expression at the end of the temporal cascade, though there are other temporal factors that also overlap in expression and thus, exert additional regulatory effects (Konstantinides et al., 2022). The experimental tools currently available limited our study of the influence of Tll on Scro, as misexpression of Tll would cause reversion to Type II NSC fate. However, DamID profiling of Tll detected binding at the *scro* locus. As Tll is typically a transcriptional repressor, it is likely that Tll has a repressive effect on Scro. This would present another classic interaction typically seen in temporal cascades, whereby a factor represses its predecessor to allow tighter temporal window boundaries.

We found that the reversion of INPs to Type II NSCs upon Scro misexpression was caused by the activation of Tll in young INPs. Tll is known to repress *ase* (Hakes and Brand, 2020), but also has the capacity to represses *ham* and *opa* (Fig. 7B). Interestingly, Scro misexpression also exerts repressive effects on Ham and Opa, though whether this repression is mediated only through the activation of Tll will have to be determined through the simultaneous misexpression of Scro and knockdown of *tll*. When *tll* is knocked down in late INPs, there is an increase in INP number as well as prolonged expression of Ham and Opa. This suggests that the termination of INP proliferation involves the inactivation of both factors. On the other hand, the role of Ham in immature INPs is to maintain INP cell identity through the sequential inactivation of *tll* through histone deacetylation (Rives-Quinto et al., 2020). Interestingly, our DamID data suggests that Tll binds to the *ham* locus, possibly repressing its expression in Type II NSCs and late INPs. The inactivation of *tll* by Ham in immature INPs suggests a cross-repressive relationship between the two. The dosage of Ham or Tll in the cell could therefore determine downstream transcriptional regulation. This highlights that similar gene regulatory networks can be used in different cell types to produce different outcomes: Tll maintains Type II NSC cell fate and must be inactivated for INP maturation, while Tll drives the termination of proliferation in late INPs. The difference in the action of Tll may be dependent on other factors, such as the changing chromatin landscape and expression of co-factors that may alter the binding activity or repressive capacity of Tll at its target loci. In optic lobe NSCs, Opa is repressed by Scro and does not overlap with the Tll temporal window. DamID data of both the INPs and optic lobe NSCs show that Tll does bind to the *opa* locus, which may be to maintain repression at this locus originally initiated by Scro. Tll binding at the *ham* locus in the optic lobe NSCs may also be evidence of its repressive activity, similar to that observed in the INPs.

Gliogenesis in both the Type II and optic lobe lineages occurs after neurogenesis, overlapping with the expression of Tll. Although its expression coincides with the production of glia, Tll only acts to impair gliogenesis in the INPs, with Scro having a more substantial role. In optic lobe NSCs, it is *gcm*, rather than Tll that primarily regulates gliogenesis (Bernardoni et al., 1999; Zhu et al., 2022). However, it remains possible that Tll has a mild effect on gliogenesis in the optic lobe, in a similar fashion to that observed in INPs. Tll is primarily characterised as a transcriptional repressor; however, Tll binds significantly to both the *repo* and *gcm* loci in Type II NSCs, so perhaps could have a context-specific role as an activator. It is also conceivable that Tll may act as a repressor to ensure that gliogenesis does not occur prematurely, while allowing a priming of the gliogenic state for a timely switch. In INPs, however, Tll binds to the *repo* locus but not to *gcm*. No significant binding was detected in the optic lobe NSCs. The expression pattern of *gcm* in the INPs and absence of binding suggests it is not regulated or activated by Tll. Akin to the optic lobe, Gcm may be the primary regulator of gliogenesis, with Tll playing a secondary role in the process of glial differentiation and maturation.

Tll expression in the INPs is also variable between DM lineages, notably absent in DMs 1 and 4. Although the INP temporal cascades between DM lineages share some of the temporal factors, there are clear differences in INP number per lineage, in the types of neuronal or glial progeny produced and in the duration of temporal windows. Although DM1 and 4 INPs do not express Tll, these DM lineages still produce glia, with DM4 producing the least Repo+ progeny out of all six lineages. Although DM1 produces Repo+ progeny in the larval brain, these are not retained in the adult (Ren et al., 2018). Interestingly, DM1 and DM4 lineages have similar INP numbers (20-30 INPs), and are the two lineages with the least amount of Grainyhead (Grh) expression (Tang et al., 2022a,b). The heterogeneity between different lineages suggests that the effect of Tll on gliogenesis may be context dependent or is regulated with other yet to be identified co-factors or temporal factors.

The functional homologue of Tll in vertebrates is TLX (NR2E1), which has conserved cell type-specific expression and protein structure (Gui et al., 2011). *Tlx* is specifically expressed in the neurogenic regions of the developing telencephalon, diencephalon, nasal placodes and retina (Islam and Zhang, 2015; Monaghan et al., 1995; Yu et al., 1994). TLX has been shown to have dynamic expression levels in the mouse brain, which peak at mid-neurogenesis then progressively diminish until birth. Loss of function of TLX in mammalian NSCs causes them to lose the capacity to self-renew and differentiate prematurely into glia (Shi et al., 2004). At later stages, TLX, in cooperation with Pax6 (orthologue of Ey), also regulates the transition of early developing NSCs into late neurogenic basal neural progenitor cells (NPCs) (Arnold et al. 2008). This is similar to how Ey and Tll in the late INPs demarcates a new window for the generation of post-mitotic progeny, transitioning to producing late-born neuronal progeny and glia. Interestingly, although there is currently little evidence for a conserved temporal patterning mechanism in mammals involving the factors we describe here, they do exhibit striking spatially restricted expression patterns in the developing mouse cerebral cortex (Fig. S3). This includes mutually exclusive expression of *Tlx* (*Nr2e1*) and *Nkx2-1*, the orthologues of Tll and Scro, respectively (Fig. S3). This suggests that the mutually repressive relationship of the two could be conserved and raises the possibility that *Drosophila* temporal factors may have been evolutionarily repurposed to establish spatial rather than temporal identity. It is conceivable that these factors each maintain their cross-regulatory relationships, which could act to create sharp expression borders in space in the same way they have been proven to do in time.

TLX has been demonstrated to have clear roles in gliogenesis in the adult mammalian central nervous system. TLX expression is present in mature astrocytes and Müller glia of the visual system, as well as in immature astrocytes prior to their migration into the inner nuclear layer (Miyawaki et al., 2004). In the subventricular zone of the adult murine cortex, TLX is expressed in both NSCs and transit amplifying progenitors (Obernier et al., 2014). TLX inactivation led to increases in the expression of OLIG2 and GFAP in NSCs, and transit amplifying precursors, respectively. This indicates that the roles of Tll/TLX in the repression of gliogenesis could well be conserved between *Drosophila* and mouse.

In summary, we have identified Tll as a terminal temporal factor in the INP temporal cascade. As a terminal temporal factor, it is activated by the preceding factor Scro and reciprocally represses it, forming a classic regulatory interaction observed in many temporal cascades. Tll also represses Opa, Ham and Ase, which all have effects on INP longevity, suggesting that the timely termination of INP proliferation requires precise regulation of these factors. In the INPs, Tll also plays a role in gliogenesis, coinciding with the temporal window during which glia are generated. The expression pattern of Tll, and conserved binding activity across the INPs and OL NSCs highlights the remarkable similarities of neural lineages in the central brain and visual system.

## MATERIALS AND METHODS

### Fly husbandry and stocks

Embryos were collected on apple juice plates supplemented with wet yeast. Adult flies and larvae were fed with standard sugar/yeast agar (SYA) food and kept at 25°C. For experiments requiring temperature shifts, larvae were reared at 18°C and shifted 29°C to inactivate the temperature-sensitive GAL80.

The following lines were used: GMR9D11 (*erm*)-GAL4 [Bloomington Drosophila Stock Centre (BDSC), BL40731), GMR12E09 (*D*)-GAL4 (BDSC, BL48510), GAL4^c855a^ (Manseau et al., 1997), *ogre*-GAL4 (BDSC, BL49340), Act5C>y^+^>GAL4 (or Ay-GAL4), UAS-GFP (BDSC, BL4411), 10X-UAS-IVS-mCD8-RFP (BDSC, BL32219), UAS-FLP (BDSC, BL4539, BL4540), UAS-tll (Kyoto DGRC, 109680), UAS-RNAi-mCherry (BDSC, BL35785), UAS-*scro*-RNAi (BDSC, BL33890) (BDSC, BL4411), UAS-tll-miRNA[s] (Lin et al., 2009), UAS-Scro (Tang et al., 2022a,b), UAS-NanoDam (Tang et al., 2022a,b), tubGAL80^ts^ (BDSC, BL7018), ase-GAL80 (Neumüller et al., 2011) and Tll-GFP (BDSC, BL30874).

### Immunohistochemistry

Larval brains were dissected in PBS and fixed in 4% formaldehyde in PBS for 20 min at room temperature. Embryos or dissected brains were washed in 0.3% Triton in PBS (PBST) for 3×5 min at room temperature. The following primary antibodies were used: chicken anti-GFP (1:2000, Abcam), guinea pig anti-Dpn (1:5000, Caygill and Brand, 2017), rabbit anti-Tll (1:300, Asian Distribution Center for Segmentation Antibodies, Kosman et al., 1998), rabbit anti-Repo (1:100,000, a gift from Benjamin Altenhein, University of Cologne, Germany), rabbit anti-Opa (1:1000, a gift from Michael Akam, University of Cambridge, UK), guinea pig anti-Dichaete (1:2000, a gift from Alex Gould, Crick Institute, UK), rat anti-Grh (1:1000, a gift from Stefan Thor, University of Queensland, Australia) and rabbit anti-Ase (1:300, a gift from Cheng-Yu Lee, University of Michigan, USA).

Antibodies were diluted in PBST and samples were incubated overnight at 4°C. Samples were washed three times for 15 min each in PBST at room temperature. Secondary antibodies (Alexa Fluor 405, 488, 546, 568 and 633 from Life Technologies; DyLight 405, Jackson Laboratories) were diluted in PBST at 1:200 concentration and incubated overnight at 4°C.

### Hybridisation chain reaction

Samples were fixed as described above and washed three times for 5 min each in PBS. Probes were designed against the coding sequence of the genes of interest: *tll* (B1), *ase* (B4), *scro* (B3) and *ham* (B5) (Molecular Instruments). 0.8 μl of probe (10 μM) was added to 200 μl of probe hybridisation buffer (PHB), which was warmed at 37°C for 30 min. The probe mix was added to the sample (replacing the PBS) and incubated at 37°C overnight. Samples were then washed with probe wash buffer (PWB) four times for 15 min each at 37°C, followed by one 5 min wash with PWB/0.3% Triton in SSC (SSCT) (1:1 ratio) and two 5 min washes in SSCT at room temperature. Amplifiers were heated at 97°C for 90 s and allowed to cool in the dark for 30 min at room temperature. For each sample, 2 μl of h1 and h2 amplifier was heated and cooled separately, then added to 100 μl amplification buffer at room temperature. This amplification mix was added to the samples (replacing the SSCT wash). Samples were incubated in the dark overnight at room temperature.

Samples were then washed four times for 15 min each with SSCT at room temperature. For immunohistochemistry, samples were washed with a SSCT/PBST gradient (i.e. 100%, 75% and 25% SSCT), with each wash lasting 5 min at room temperature. Samples were then washed three times for 5 min each in PBST then stained as described above. Samples were mounted on glass slides using SlowFade Diamond (Thermo Fisher Scientific).

### Image acquisition and processing

Images were acquired using a Leica DM6 CS Confocal Microscope. Quantifications of cell numbers, adjustments to brightness and contrast were carried out using Fiji (ImageJ). Figures were made using Adobe Illustrator. Quantitative data and statistical analysis as represented through graphs were made using Prism.

### NanoDam and DamID profiling

#### Tll TaDa in INPs

Tll binding in INPs was profiled by driving the UAS-Tll-Dam construct using D-GAL4 and a temperature-sensitive *tub*-GAL80 to control onset of expression. *D*-GAL4 driving UAS-Dam-alone was used as a control. Larvae with the genotype *D*-GAL4>UAS-Tll-Dam or UAS-Dam-alone were placed at 18°C for 6 days then shifted to 29°C for 16 h and subsequently dissected in PBS.

#### Tll NanoDam in the neuroepithelium and optic lobe NSCs

Endogenous Tll-GFP binding in the neuroepithelium was profiled by driving UAS-NanoDam with c855a-GAL4 and *ase*-GAL80 to prevent expression in the optic lobe NSCs. A temperature-sensitive GAL80 was also used to modulate NanoDam induction. c855a-GAL4, *ase*-GAL80, tub-GAL80^ts^>UAS-NanoDam without Tll-GFP was used as a control. Larvae were kept at 18°C for 4 days then shifted to 29°C for 16 h before dissection in PBS. For optic lobe NSC profiling, *ogre*-GAL4 was used to drive UAS-NanoDam in the presence of Tll-GFP, with a temperature-sensitive GAL80 to control onset of NanoDam induction. *ogre*-GAL4, *tub*-GAL80^ts^>UAS-NanoDam without Tll-GFP was used as a control. Larvae were placed at 18°C for 6 days and shifted to 29°C for 16 h before dissection.

30-50 brains were dissected for each condition and control, with three or four biological replicates per profiling experiment. After dissection, excess PBS was removed and samples were stored at −80°C for later processing. Genomic library construction and analysis of DamID-seq data was performed as described by Tang et al. (2022a,b) STAR protocols.

### Motif enrichment analysis

For motif enrichment analysis, the STREME (version 5.5.9) web version from the MEME tools suite was used (Bailey, 2021). For each cell type, a .BED file containing the identified Tll-binding peaks was compared to Dam only binding peaks, which served as control sequences. The top 10 sequences that were statistically significant (*P*<0.05) were then compared against known motifs compiled in *Drosophila* databases using Tomtom (version 5.5.9) (Gupta et al., 2007).

## Supplementary Material

10.1242/develop.205703_sup1 Supplementary information

Table S1. Enriched binding motifs identified in Type II NSCs, INPs and OL NSCs.

## References

[DEV205703C1] Abdusselamoglu, M. D., Eroglu, E., Burkard, T. R. and Knoblich, J. A. (2019). The transcription factor odd-paired regulates temporal identity in transit-amplifying neural progenitors via an incoherent feed-forward loop. *eLife* 8, e46566. 10.7554/eLife.46566 PMC6645715 31329099

[DEV205703C2] Alsiö, J. M., Tarchini, B., Cayouette, M. and Livesey, F. J. (2013). Ikaros promotes early-born neuronal fates in the cerebral cortex. *Proc. Natl. Acad. Sci. USA* 110, E716-E725. 10.1073/pnas.1215707110 PMC3581915 23382203

[DEV205703C43] Arnold, S. J., Huang, G. J., Cheung, A. F., Era, T., Nishikawa, S., Bikoff, E. K., Molnár, Z., Robertson, E. J. and Groszer, M. (2008). The T-box transcription factor Eomes/Tbr2 regulates neurogenesis in the cortical subventricular zone. *Genes Dev.* 22, 2479-2484. 10.1101/gad.475408 PMC2546697 18794345

[DEV205703C3] Bailey, T. L. (2021). STREME: accurate and versatile sequence motif discovery. *Bioinformatics* 37, 2834-2840. 10.1093/bioinformatics/btab203 PMC8479671 33760053

[DEV205703C4] Bayraktar, O. A. and Doe, C. Q. (2013). Combinatorial temporal patterning in progenitors expands neural diversity. *Nature* 498, 449-455. 10.1038/nature12266 PMC3941985 23783519

[DEV205703C5] Bello, B. C., Izergina, N., Caussinus, E. and Reichert, H. (2008). Amplification of neural stem cell proliferation by intermediate progenitor cells in Drosophila brain development. *Neural Dev.* 3, 5. 10.1186/1749-8104-3-5 PMC2265709 18284664

[DEV205703C6] Bernardoni, R., Kammerer, M., Vonesch, J.-L. and Giangrande, A. (1999). Gliogenesis depends on glide/gcm through asymmetric division of neuroglioblasts. *Dev. Biol.* 216, 265-275. 10.1006/dbio.1999.9511 10588877

[DEV205703C7] Boone, J. Q. and Doe, C. Q. (2008). Identification of Drosophila type II neuroblast lineages containing transit amplifying ganglion mother cells. *Dev. Neurobiol.* 68, 1185-1195. 10.1002/dneu.20648 PMC2804867 18548484

[DEV205703C8] Bowman, S. K., Rolland, V., Betschinger, J., Kinsey, K. A., Emery, G. and Knoblich, J. A. (2008). The tumor suppressors brat and numb regulate transit-amplifying neuroblast lineages in Drosophila. *Dev. Cell* 14, 535-546. 10.1016/j.devcel.2008.03.004 PMC2988195 18342578

[DEV205703C9] Caviness, V. S. (1982). Neocortical histogenesis in normal and reeler mice: a developmental study based upon [3H]thymidine autoradiography. *Dev. Brain Res.* 4, 293-302. 10.1016/0165-3806(82)90141-9 7104762

[DEV205703C44] Caygill, E. E. and Brand, A. H. (2017). miR-7 buffers differentiation in the developing Drosophila visual system. *Cell Rep.* 20, 1255-1261. 10.1016/j.celrep.2017.07.047 PMC5561169 28793250

[DEV205703C10] Doe, C. Q. (2017). Temporal patterning in the Drosophila CNS. *Annu. Rev. Cell Dev. Biol.* 33, 219-240. 10.1146/annurev-cellbio-111315-125210 28992439

[DEV205703C11] Egger, B., Boone, J. Q., Stevens, N. R., Brand, A. H. and Doe, C. Q. (2007). Regulation of spindle orientation and neural stem cell fate in the Drosophila optic lobe. *Neural Dev.* 2, 1. 10.1186/1749-8104-2-1 PMC1779784 17207270

[DEV205703C12] Egger, B., Gold, K. S. and Brand, A. H. (2010). Notch regulates the switch from symmetric to asymmetric neural stem cell division in the Drosophila optic lobe. *Development* 137, 2981-2987. 10.1242/dev.051250 PMC2926952 20685734

[DEV205703C13] Eroglu, E., Burkard, T. R., Jiang, Y., Saini, N., Homem, C. C. F., Reichert, H. and Knoblich, J. A. (2014). SWI/SNF complex prevents lineage reversion and induces temporal patterning in neural stem cells. *Cell* 156, 1259-1273. 10.1016/j.cell.2014.01.053 24630726

[DEV205703C14] Gui, H., Li, M.-L. and Tsai, C.-C. (2011). A tale of tailless. *Dev. Neurosci.* 33, 1-13. 10.1159/000321585 PMC3080779 21124006

[DEV205703C15] Guillermin, O., Perruchoud, B., Sprecher, S. G. and Egger, B. (2015). Characterization of tailless functions during Drosophila optic lobe formation. *Dev. Biol.* 405, 202-213. 10.1016/j.ydbio.2015.06.011 26111972

[DEV205703C16] Gupta, S., Stamatoyannopoulos, J. A., Bailey, T. L. and Noble, W. S. (2007). Quantifying similarity between motifs. *Genome Biol.* 8, R24. 10.1186/gb-2007-8-2-r24 PMC1852410 17324271

[DEV205703C17] Hakes, A. E. and Brand, A. H. (2020). Tailless/TLX reverts intermediate neural progenitors to stem cells driving tumourigenesis via repression of asense/ASCL1. *eLife* 9, e53377. 10.7554/eLife.53377 PMC7058384 32073402

[DEV205703C18] Holguera, I. and Desplan, C. (2018). Neuronal specification in space and time. *Science* 362, 176-180. 10.1126/science.aas9435 PMC6368964 30309944

[DEV205703C19] Islam, M. M. and Zhang, C.-L. (2015). TLX: a master regulator for neural stem cell maintenance and neurogenesis. *Biochim. Biophys. Acta* 1849, 210-216. 10.1016/j.bbagrm.2014.06.001 PMC4265312 24930777

[DEV205703C20] Komori, H., Xiao, Q., Janssens, D. H., Dou, Y. and Lee, C.-Y. (2014). Trithorax maintains the functional heterogeneity of neural stem cells through the transcription factor Buttonhead. *eLife* 3, e03502. 10.7554/eLife.03502 PMC4221733 25285447

[DEV205703C21] Konstantinides, N., Holguera, I., Rossi, A. M., Escobar, A., Dudragne, L., Chen, Y.-C., Tran, T. N., Martínez Jaimes, A. M., Özel, M. N., Simon, F. et al. (2022). A complete temporal transcription factor series in the fly visual system. *Nature* 604, 316-322. 10.1038/s41586-022-04564-w PMC9074256 35388222

[DEV205703C22] Kosman, D., Small, S. and Reinitz, J. (1998). Rapid preparation of a panel of polyclonal antibodies to Drosophila segmentation proteins. *Dev. Gene. Evol.* 208, 290-294. 10.1007/s004270050184 9683745

[DEV205703C23] Li, X., Erclik, T., Bertet, C., Chen, Z., Voutev, R., Venkatesh, S., Morante, J., Celik, A. and Desplan, C. (2013). Temporal patterning of Drosophila medulla neuroblasts controls neural fates. *Nature* 498, 456-462. 10.1038/nature12319 PMC3701960 23783517

[DEV205703C24] Lin, S., Huang, Y. and Lee, T. (2009). Nuclear receptor unfulfilled regulates axonal guidance and cell identity of Drosophila mushroom body neurons. *PLoS ONE* 4, e8392. 10.1371/journal.pone.0008392 PMC2793019 20027309

[DEV205703C25] Manseau, L., Baradaran, A., Brower, D., Budhu, A., Elefant, F., Phan, H., Philp, A. V., Yang, M., Glover, D., Kaiser, K. et al. (1997). GAL4 enhancer traps expressed in the embryo, larval brain, imaginal discs, and ovary of drosophila. *Dev. Dyn.* 209, 310-322. 10.1002/(SICI)1097-0177(199707))209:3<310::AID-AJA6>3.0.CO;2-L 9215645

[DEV205703C26] Marshall, O. J. and Brand, A. H. (2015). damidseq_pipeline: an automated pipeline for processing DamID sequencing datasets. *Bioinformatics* 31, 3371-3373. 10.1093/bioinformatics/btv386 PMC4595905 26112292

[DEV205703C27] Marshall, O. J., Southall, T. D., Cheetham, S. W. and Brand, A. H. (2016). Cell-type-specific profiling of protein-DNA interactions without cell isolation using targeted DamID with next-generation sequencing. *Nat. Protoc.* 11, 1586-1598. 10.1038/nprot.2016.084 27490632

[DEV205703C45] Miyawaki, T., Uemura, A., Dezawa, M., Yu, R. T., Ide, C., Nishikawa, S., Honda, Y., Tanabe, Y. and Tanabe, T. (2004). Tlx, an orphan nuclear receptor, regulates cell numbers and astrocyte development in the developing retina. *J. Neurosci.* 24, 8124-8134. 10.1523/JNEUROSCI.2235-04.2004 PMC6729803 15371513

[DEV205703C28] Monaghan, A. P., Grau, E., Bock, D. and Schütz, G. (1995). The mouse homolog of the orphan nuclear receptor *tailless* is expressed in the developing forebrain. *Development* 121, 839-853. 10.1242/dev.121.3.839 7720587

[DEV205703C29] Neumüller, R. A., Richter, C., Fischer, A., Novatchkova, M., Neumüller, K. G. and Knoblich, J. A. (2011). Genome-wide analysis of self-renewal in drosophila neural stem cells by transgenic RNAi. *Cell Stem Cell* 8, 580-593. 10.1016/j.stem.2011.02.022 PMC3093620 21549331

[DEV205703C30] Obernier, K., Tong, C. K. and Alvarez-Buylla, A. (2014). Restricted nature of adult neural stem cells: re-evaluation of their potential for brain repair. *Front. Neurosci.* 8, 162. 10.3389/fnins.2014.00162 PMC4060730 24987325

[DEV205703C31] Ren, Q., Awasaki, T., Wang, Y.-C., Huang, Y.-F. and Lee, T. (2018). Lineage-guided Notch-dependent gliogenesis by Drosophila multi-potent progenitors. *Development* 145, dev160127. 10.1242/dev.160127 PMC6031320 29764857

[DEV205703C32] Rives-Quinto, N., Komori, H., Ostgaard, C. M., Janssens, D. H., Kondo, S., Dai, Q., Moore, A. W. and Lee, C.-Y. (2020). Sequential activation of transcriptional repressors promotes progenitor commitment by silencing stem cell identity genes. *eLife* 9, e56187. 10.7554/eLife.56187 PMC7728440 33241994

[DEV205703C33] Shi, Y., Chichung Lie, D., Taupin, P., Nakashima, K., Ray, J., Yu, R. T., Gage, F. H. and Evans, R. M. (2004). Expression and function of orphan nuclear receptor TLX in adult neural stem cells. *Nature* 427, 78-83. 10.1038/nature02211 14702088

[DEV205703C34] Southall, T. D., Gold, K. S., Egger, B., Davidson, C. M., Caygill, E. E., Marshall, O. J. and Brand, A. H. (2013). Cell-type-specific profiling of gene expression and chromatin binding without cell isolation: assaying RNA Pol II occupancy in neural stem cells. *Dev. Cell* 26, 101-112. 10.1016/j.devcel.2013.05.020 PMC3714590 23792147

[DEV205703C35] Stam, F. J., Hendricks, T. J., Zhang, J., Geiman, E. J., Francius, C., Labosky, P. A., Clotman, F. and Goulding, M. (2012). Renshaw cell interneuron specialization is controlled by a temporally restricted transcription factor program. *Development* 139, 179-190. 10.1242/dev.071134 PMC3231776 22115757

[DEV205703C36] Tang, J. L. Y., Hakes, A. E., Krautz, R., Suzuki, T., Contreras, E. G., Fox, P. M. and Brand, A. H. (2022a). NanoDam identifies Homeobrain (ARX) and Scarecrow (NKX2.1) as conserved temporal factors in the Drosophila central brain and visual system. *Dev. Cell* 57, 1193-1207.e7. 10.1016/j.devcel.2022.04.008 PMC9616798 35483359

[DEV205703C37] Tang, J. L. Y., Krautz, R., Llorà-Batlle, O., Hakes, A. E., Fox, P. M. and Brand, A. H. (2022b). In vivo, genome-wide profiling of endogenously tagged chromatin-binding proteins with spatial and temporal resolution using NanoDam in Drosophila. *STAR Protoc.* 3, 101788. 10.1016/j.xpro.2022.101788 PMC9636480 36345375

[DEV205703C38] Telley, L., Agirman, G., Prados, J., Amberg, N., Fièvre, S., Oberst, P., Bartolini, G., Vitali, I., Cadilhac, C., Hippenmeyer, S. et al. (2019). Temporal patterning of apical progenitors and their daughter neurons in the developing neocortex. *Science* 364, eaav2522. 10.1126/science.aav2522 31073041

[DEV205703C39] Xie, Y., Li, X., Zhang, X., Mei, S., Li, H., Urso, A. and Zhu, S. (2014). The Drosophila Sp8 transcription factor Buttonhead prevents premature differentiation of intermediate neural progenitors. *eLife* 3, e03596. 10.7554/eLife.03596 PMC4221738 25285448

[DEV205703C40] Young, R. W. (1985). Cell differentiation in the retina of the mouse. *Anat. Rec.* 212, 199-205. 10.1002/ar.1092120215 3842042

[DEV205703C41] Yu, R. T., McKeown, M., Evans, R. M. and Umesono, K. (1994). Relationship between Drosophila gap gene tailless and a vertebrate nuclear receptor Tlx. *Nature* 370, 375-379. 10.1038/370375a0 8047143

[DEV205703C42] Zhu, H., Zhao, S. D., Ray, A., Zhang, Y. and Li, X. (2022). A comprehensive temporal patterning gene network in Drosophila medulla neuroblasts revealed by single-cell RNA sequencing. *Nat. Commun.* 13, 1247. 10.1038/s41467-022-28915-3 PMC8913700 35273186

