# Supplementary material for: Tailless terminates the neural stem cell temporal cascade in both the optic lobe and central brain
Source: Development. 2026 Sep 14;153(17):dev205703. doi: 10.1242/dev.205703 (PMC13615674; doi:10.1242/dev.205703)
Supplement: Supplementary file 1 [file develop-153-205703-s1.pdf]

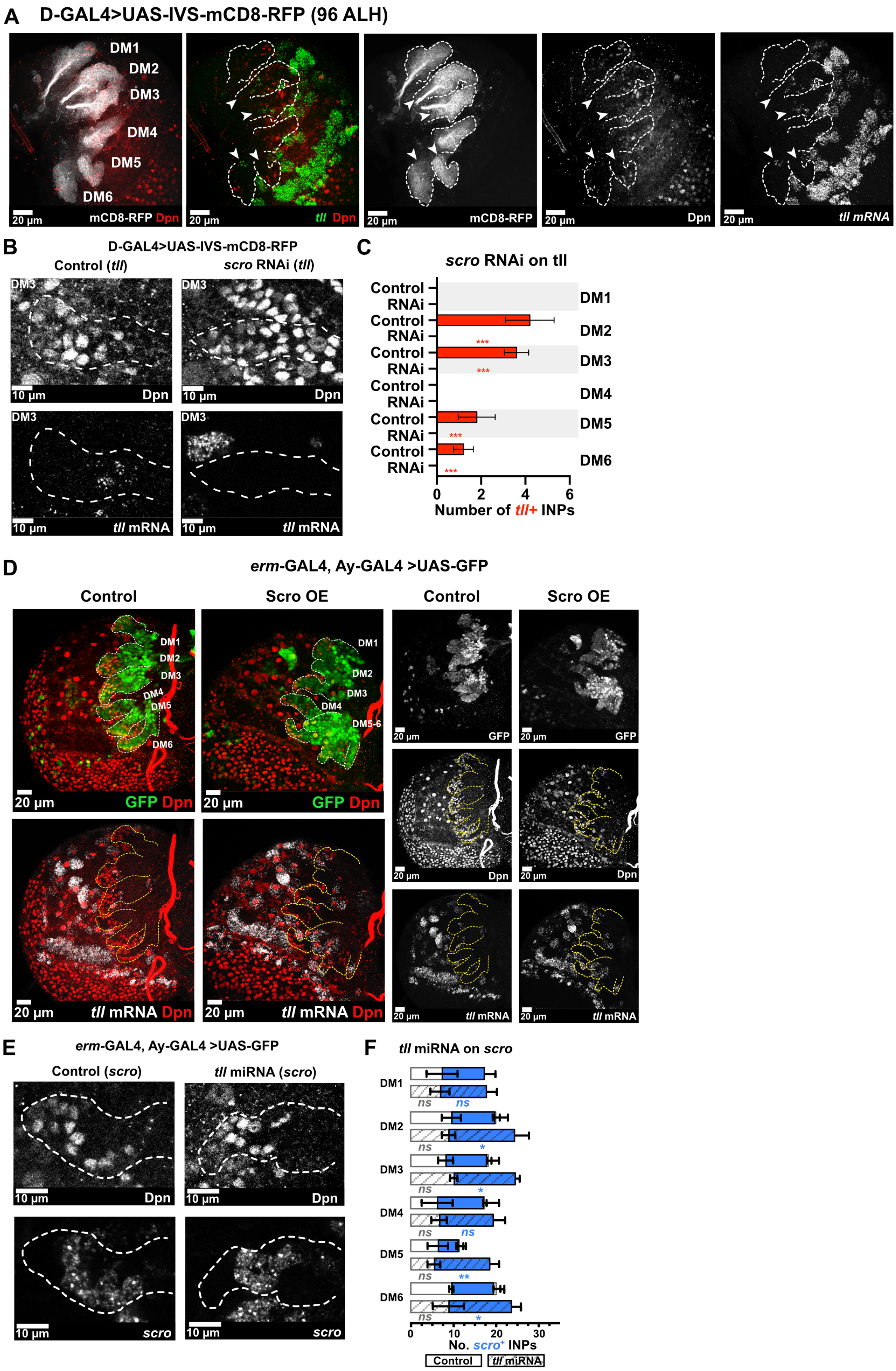

Development • Supplementary information

Fig. S1. Expression of Tll in late INPs.

(A) Max intensity confocal images showing *tll* expression across all six DM lineages. Dotted lines in overlay and single channel show boundaries of lineages labelled by *D*-GAL4. White arrows show lineages where *tll* expression was detected.
(B) Single slice confocal images of the effect of *scro* knockdown of tll expression split by channels, corresponding to Figure 1E.
(C) Quantification of the number of *tll*-positive INPs upon *scro* knockdown, split by DM lineage.
(D) Single slice confocal images showing the *erm*-GAL4>UAS-Scro phenotype across all DM lineages (dotted outlines), with respective single channel images.
(E) Single slice confocal images of the effect of *scro* knockdown of tll expression split by channels, corresponding to Figure 1G.
(F) Quantification of the number of *scro*-positive INPs (blue) out of the total number of INPs upon *tll* knockdown, split by DM lineage. Significance values are shown for the number of *scro*-positive (blue) and *scro*-negative (grey) INPs.

All larval brains were staged to 96 hours after larval hatching (ALH). Graphs show mean ± s.d. Statistical significance was tested with the Mann Whitney U test, p-values are represented by: *** ($p<0.001$), ** ($p<0.005$), * ($p<0.05$) and n.s ($p>0.05$).

Development • Supplementary information

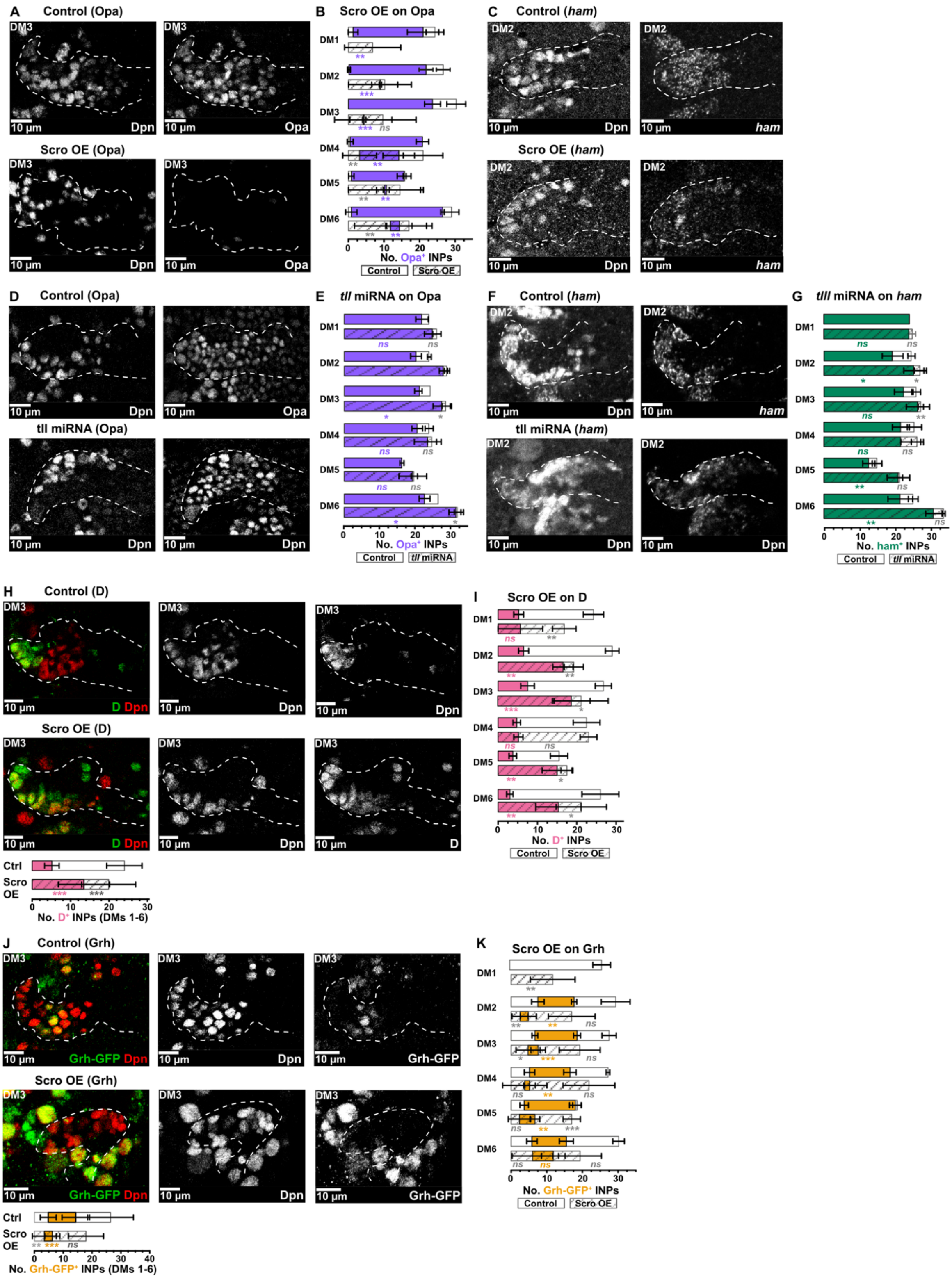

Development • Supplementary information

Fig. S2. Overexpression of Scro on Opa, D and Grh expression.

(A) Single slice confocal images showing the overexpression of Scro leads to a reduction in Opa expression and INP number. Lineages (dotted lines) labelled by *D*-GAL4>UAS-IVS-mCD8-RFP. Grayscale images show Opa and Dpn channels separately.

(B) Graph show the number of Opa-expressing cells out of the total number INPs counted per DM lineage.

(C) Single slice confocal images showing overexpression of Scro leads to reduced ham expression. Lineages (dotted lines) labelled by *erm*-GAL4, Ay-GAL4>UAS-GFP. Grayscale images show *ham* and Dpn channels separately.

(D) Single slice confocal images showing knockdown of *tll* leads to prolonged Opa expression. Lineages (dotted lines) labelled by *D*-GAL4>UAS-IVS-mCD8-RFP. Grayscale images show Opa and Dpn channels separately.

(E) Graph show the number of Opa-expressing cells out of the total number INPs counted per DM lineage.

(F) Single slice confocal images showing knockdown of *tll* leads to prolonged *ham* expression. Lineages (dotted lines) labelled by *D*-GAL4>UAS-IVS-mCD8-RFP. Grayscale images show *ham* and Dpn channels separately.

(G) Graph show the number of *ham*-expressing cells out of the total number INPs counted per DM lineage.

(H) Single slice confocal images showing overexpression of Scro leads to expansion of the early D window. A late temporal factor unexpectedly causes expanded expression of the early temporal factor Dichaete. This is explained by the repression of Odd-paired, which normally represses Dichaete (Abdusselamoglu et al. 2019). Lineages (dotted lines) labelled by *D*-GAL4>UAS-IVS-mCD8-RFP. Number of D-positive cells out of the total number of INPs are shown as an average across DMs 1-6. Grayscale images show D and Dpn channels separately.

Development • Supplementary information

(I) Graph show the number of D-expressing cells out of the total number INPs counted per DM lineage, n=6 brains.
(J) Single slice confocal images showing overexpression of Scro leads to a restriction of the Grh window. Lineages (dotted lines) labelled by *D*-GAL4>UAS-IVS-mCD8-RFP. Number of Grh-positive cells out of the total number of INPs are shown as an average across DMs 1-6. Grayscale images show Grh and Dpn channels separately.
(K) Graph show the number of Grh-expressing cells out of the total number INPs counted per DM lineage, n=6 brains.

All larval brains were staged to 96 hours after larval hatching (ALH). Graphs show mean ± s.d. Statistical significance was tested with the Mann Whitney U test, p-values are represented by: *** ($p<0.001$), ** ($p<0.005$), * ($p<0.05$) and n.s ($p>0.05$).

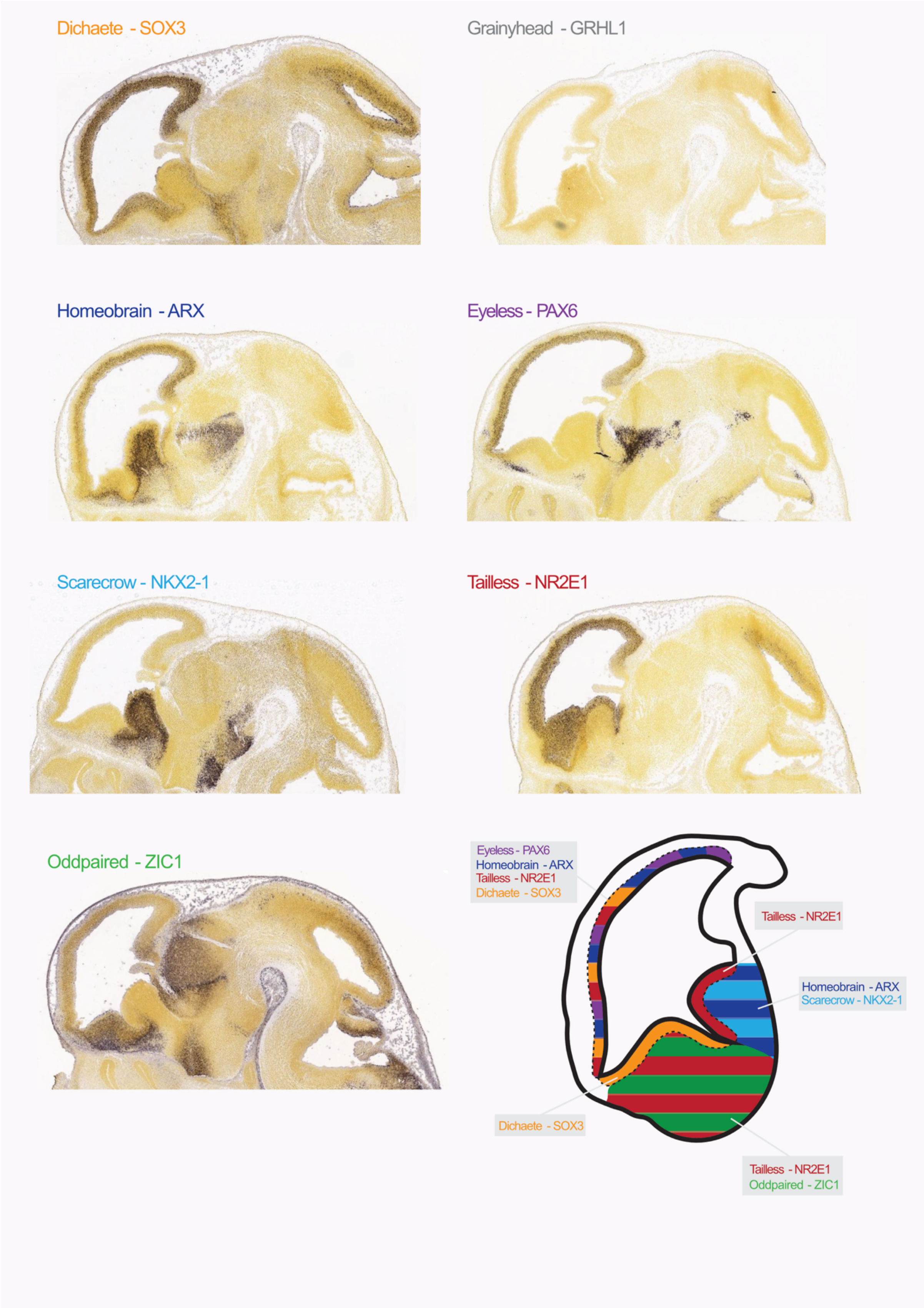
Dichaete - SOX3
Grainyhead - GRHL1
Homeobrain - ARX
Eyeless - PAX6
Scarecrow - NKX2-1
Tailless - NR2E1
Oddpaired - ZIC1
Eyeless - PAX6
Homeobrain - ARX
Tailless - NR2E1
Dichaete - SOX3
Tailless - NR2E1
Homeobrain - ARX
Scarecrow - NKX2-1
Dichaete - SOX3
Tailless - NR2E1
Oddpaired - ZIC1

Fig. S3. *In situ* hybridisation data from E13.5 mouse brain acquired from the Allen developing mouse brain atlas showing spatially restricted expression of *Drosophila* temporal factor orthologues. Schematic depicts the spatial expression patterns of these temporal factor orthologues in relation to one another.

Table S1. Enriched binding motifs identified in Type II NSCs, INPs and OL NSCs.

Available for download at
https://journals.biologists.com/dev/article-lookup/doi/10.1242/dev.205703#supplementary-data
